# Association of low birthweight and small for gestational age with maternal ferritin levels: A retrospective cohort study in China

**DOI:** 10.3389/fnut.2022.1002702

**Published:** 2022-10-10

**Authors:** Yun Tao, Jiawei Kang, Juan Liu, Jie Duan, Fang Wang, Yue Shi, Yujuan Li, Cheng Wang, Dan Xu, Xinlan Qu, Juanjuan Guo, Jianhong Ma, Yuanzhen Zhang

**Affiliations:** ^1^Department of Obstetrics and Gynecology, Zhongnan Hospital of Wuhan University, Wuhan, China; ^2^Hubei Clinical Research Center for Prenatal Diagnosis and Birth Health, Wuhan, China; ^3^Wuhan Clinical Research Center for Reproductive Science and Birth Health, Wuhan, China; ^4^Information Center, Zhongnan Hospital of Wuhan University, Wuhan, China

**Keywords:** ferritin, cohort study, pregnancy nutrition, low birthweight, small for gestational age

## Abstract

**Background:**

Birthweight have profound impacts on health status throughout lifetime, however, the relationship between maternal ferritin level in pregnancy and birthweight of the newborn remains controversial.

**Objective:**

This retrospective cohort research was to analyze the association between maternal ferritin levels during pregnancy with birthweight outcomes, primarily for low birthweight (LBW) and small for gestational age (SGA).

**Methods:**

Newborns weighing lower than 2,500 grams were defined as LBW. SGA is defined as birthweight lower than the 10^th^ percentile of the distribution of newborns' birthweight of the same gestational age. Multivariable logistic regressions have been used to explore the association of maternal ferritin levels and birthweight related outcomes, in which the ferritin concentration was logarithm transformed in the model. We further used restricted cubic spline models to explore linear/non-linear dose–response manners of ferritin level and birthweight outcomes.

**Results:**

A total of 3,566 pregnant women were included in the study. In the results of the present study, we observed that maternal ferritin levels were linearly associated with the risk of LBW (*p-*trend = 0.005) and SGA (*p-*trend = 0.04), with the adjusted odds ratios (ORs) of 1.78 (95% CI 1.37–2.32) for LBW and 1.87 (95% CI 1.38–2.54) for SGA with an increase in Ln-ferritin concentrations per unit. The adjusted ORs across quartiles of ferritin levels were 2.14 (95% CI 1.03–4.47) for Quartile 2, 3.13 (95% CI 1.47–6.69) for Quartile 3, and 3.63 (95% CI 1.52–8.68) for Quartile 4 for LBW. The adjusted ORs of LBW and SGA among women using supplemental iron were 0.56 (95% CI 0.38, 0.85) and 0.65 (95% CI 0.40, 1.05) compared with non-users, respectively.

**Conclusions:**

Our findings found a linear dose–response relationship between ferritin levels and an increased risk of poor birthweight outcomes, suggesting that maternal ferritin level during pregnancy may provide an additional predictor for differentiating poor birthweight related outcomes. Further exploration should be conducted to ensure maternal ferritin thresholds and iron supplement doses.

## Introduction

Birthweight is the key to evaluating fetal growth (1). Poor birthweight due to intrauterine growth and development limitations may contribute to adverse birth outcomes. Low birthweight (LBW) was the major cause of morbidity and mortality in newborns (1). Low birthweight is a global public health problem, which involves a wide range and has short-term and also long-term impact on the early growth and development of individuals, as well as the occurrence of chronic diseases in adolescence and adulthood. The prevalence of low birthweight remains high, this phenomenon adds a heavy burden to the medical and health system (2). The incidence rate of low birthweight reflects the nutritional status of pregnant women and fetuses in a place or country, as well as the status and the quality of maternal and child's health care work. Therefore, fetal weight undergrowth is an important neonatal outcome, and it is important to identify its potential influencing factors to prevent adverse health effects throughout life time.

The nutrition status of pregnant woman during pregnancy critically determines the nutrition of the fetus and contributes significantly to the health of the fetus and newborn. Serum ferritin is a protein that indicates the level of iron reserve in the body (3). Poor ferritin level is a common nutritional problem during pregnancy and is associated with inadequate intake anemia or certain chronic diseases (4). Ferritin itself has important physiological activities which is not only a carrier of iron (3). Iron, on the other hand, is a powerful pro-oxidant that catalyzes the production of reactive oxidative species (ROS) (5). Excess iron intake can lead to impaired placenta function because of oxidative damage (4). Existing epidemiological evidence demonstrates that higher serum ferritin is associated with an increased risk of LBW in late pregnancy (6). Some researchers found that both low and high ferritin levels measured in pregnancy were add risks to the occurrence of LBW and small for gestational age (SGA) among newborns with small sample size (7), and hypothesized the relationship between iron stores and total health risk would be U-shaped, similar to most other nutrients (8). Obstetricians pay attention to ferritin levels during pregnancy, pregnant women are encouraged to take iron supplements when ferritin levels are low (9). In China, the prevalence of anemia in non-pregnant women was 17.4%, in women during the first trimester was 21.6%, and in women during the third trimester was 10.5% (10). As a result, the spontaneous go to pharmacy for iron contained supplements by pregnant women is common in the context of high anemia rates. Because pregnancy is an especially sensitive period, the type of food consumed by pregnant women and the independent choice of various doses of nutritional supplements will greatly affect the level of ferritin during pregnancy.

In conclusion, the results of previous researches on iron status and the risk of LBW have no consistent conclusion. Additionally, there is no evidence of the dose–response relationship between serum ferritin concentration and birthweight outcomes. Furthermore, research on iron overload that utilizes large sample sizes is limited among the Chinese population. This study was conducted to investigate the relationship between maternal serum ferritin levels and birthweight of newborns, to indicate the possible outcome risk caused by iron nutrition status, to provide a scientific basis for a scientific diet, nutritional supplements, or pregnancy care.

## Methods

### Study population

This retrospective cohort study was performed at the Zhongnan Hospital of Wuhan University, China. The medical information of pregnant women who delivered in Zhongnan Hospital of Wuhan University from January 2014 to September 2021 were retrospectively reviewed, and information on pregnant women who admitted to the hospital for prenatal examinations was also collected. Inclusion criteria: (1) ≥18 years old; (2) the results of serum ferritin test during pregnancy were available; (3) the woman delivered a single fetus in our hospital; and (4) the data were complete. Exclusion criteria: (1) multiple births, stillbirths, lost visitors; (2) lack of information on newborn birthweight. This study applied for informed consent exemption and was approved by the Ethics Committee of Zhongnan Hospital of Wuhan University (Approval No.:2022141K).

### Assessment of serum ferritin and Fe supplemental use

Maternal blood samples were tested for serum ferritin by the hospital laboratory. The serum ferritin concentration was detected by the chemiluminescence method performed on Abbott^TM^ 3517. In order to ensure the precision and accuracy of ferritin detection in clinical laboratory, we conduct inner-laboratory quality control at two concentration levels daily. At the same time, the laboratory participated in the inter-laboratory evaluation plan organized by the Clinical Laboratory Center of the National Health Commission and the Clinical Laboratory Center of Hubei Provincial Health Commission to ensure that the results were comparable. All the ferritin data was extracted from the patient's first ferritin test during pregnancy in our hospital, the information of Fe supplemental use was from medical order in hospital information service.

### Birthweight outcomes

Gestational age was obtained from medical records as the number of days between delivery date and last menstrual period. Preterm birth was referring to a single live birth occurring at 36 weeks of gestation or earlier (11). Newborns weighing lower than 2,500 grams were defined as LBW (12). According to a global reference (13), SGA is defined as birthweight lower than the 10^th^ percentile of the distribution of newborns' birthweight of the same gestational age. Severe SGA was referring to birthweight lower than the 5^th^ percentile of the distribution of birth weight in the same week, and mild SGA was defined as 5^th^−9^th^ percentile of the birthweight distribution in the same week.

### Covariable

Maternal demographic and socioeconomic characteristics and lifestyle information were standardized and collected by trained nurses. The gestational weeks at the test time were also reviewed. Medical records on delivery (e.g., parity, gestational age, and infant gender) and complications of pregnancy (gestational hypertension and gestational diabetes mellitus) during pregnancy or diagnosed on admission were surveyed and reviewed by experienced obstetricians.

### Statistical analysis

The median value of each ferritin quartile was as a continuous variable in adjusted models for *p*-trend values. For outliers (exceeding the mean ± 3SD), the measured ferritin concentrations were replaced with the mean ± 3SD. Multivariable logistic regression models were used to analyze odds ratios (ORs) of birthweight outcomes across increasing quartiles of serum ferritin concentrations. Furthermore, we explored the dose–response relationships between serum ferritin levels and the risk of birthweight outcomes by restricted cubic spline models (14). Knots were placed at the 25th, 50th, 75th, and 95th percentiles of the ferritin distribution, and the reference value was set at the 50th percentile. All models were adjusted for potential confounders: maternal age at delivery (continuous), pre-pregnancy BMI (< 18.5, 18.5–23.9, 24.0–27.9, ≥28.0), hypertension (yes or no), education (≤ middle school, high school, ≥college), gestational diabetes mellitus (yes or no) and neonatal gender (male or female). Missing data of variables were categorized into an additional group to avoid losing participants caused by missing values of confounders. Stratified analyses were performed by including serum ferritin concentrations and corresponding pregnancy trimester at test time in the logistic regression models. We have conducted sensitivity analyses to verify the robustness of the results, we re-performed the analysis which limited to women freed of hypertensive disorders or gestational diabetes during the pregnancy. To further examine the combined association of ferritin concentrations and Fe supplement use with LBW, participants were stratified into three groups based on ferritin concentrations classified as low (tertile 1, ≤ 15.4 ng/mL), medium (tertile 2, 15.4–38.9 ng/mL), and high (tertile 3, ≥38.9 ng/mL), we conducted logistic regression to estimate ORs of LBW with non-users in the medium ferritin group as a reference. We performed statistical analyses by SAS 9.4.

## Results

**Figure 1:**
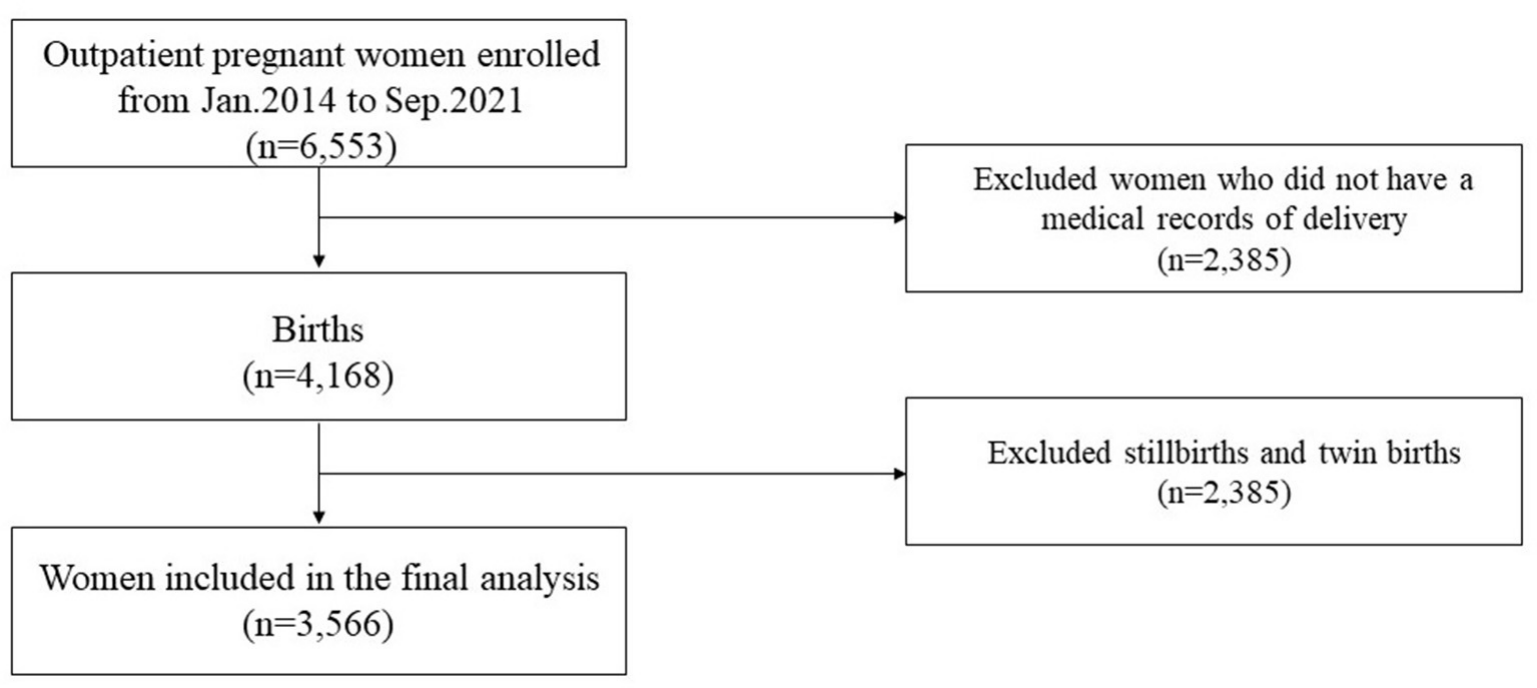
Study flowchart.

In this retrospective cohort study, 6,553 women had maternal ferritin test records before delivery who also had an admission record as an outpatient at the hospital because of “pregnancy” related diagnosis. Two thousand and eighty five women who did not have a medical record of delivery were excluded from the study. Twenty two stillbirths and 580 women who had multiple deliveries were also excluded from this analysis (Figure 1). Finally, the present study including 3,566 pregnant women in the analyses. The median (range) serum ferritin concentration in the studied population was 24.33 (< 1–793.07) ng/mL, 1,146 of them reported iron supplement use. Characteristics according to quartiles of maternal ferritin concentrations are shown in Table 1.

**Table 1:** Basic characteristics according to quartiles of maternal ferritin concentrations.

	**All individuals (*n* = 3,566)**	**Quartiles of ferritin concentration**			
		**Q1 (*n* = 891) ≤ 12.10 ng/mL**	**Q2 (*n* = 892) 12.10–24.35 ng/mL**	**Q3 (*n* = 892) 24.35–50.61 ng/mL**	**Q4 (*n* = 891) ≥50.61 ng/mL**
**Maternal characteristic**					
**Age at delivery, y**					
Mean (SD)	31.2 (4.22)	30.84 (4.22)	31.11 (4.33)	31.14 (4.18)	30.98 (4.17)
Median (range)	30 (18–53)	30 (18–49)	31 (18–53)	30 (21–50)	30 (21–46)
≤ 25	237 (6.6%)	73 (8.2%)	58 (6.5%)	50 (5.6%)	56 (6.2%)
26–29	1,191 (33.4%)	295 (33.1%)	295 (33.1)	283 (31.7%)	318 (35.7%)
30–34	1,426 (40.0%)	357 (40.1%)	355 (39.8%)	373 (41.8%)	341 (38.3%)
≥35	712 (20.0%)	166 (18.6%)	184 (20.6%)	186 (20.9%)	176 (19.8%)
**Pre-pregnancy BMI, kg/m** ^**2**^					
Mean (SD)	21.34 (3.09)	21.00 (2.73)	21.22 (2.96)	21.43 (3.19)	21.65 (3.37)
Median (range)	20.8 (13.4–49.4)	20.6 (13.7–39.8)	20.8 (14.6–45.1)	21.0 (15.1–49.4)	21.1 (13.4–38.3)
< 18.5	422 (11.8%)	104 (11.7%)	103 (11.5%)	103 (11.5%)	112 (12.5%)
18.5–23.9	1,937 (54.3%)	459 (51.5%)	481 (53.9%)/	504 (56.5%)	493 (55.3%)
24.0–27.9	349 (9.8%)	72 (8.1%)	76 (8.5%)	92 (10.3%)	109 (12.2%)
≥28.0	98 (2.8%)	13 (1.5%)	14 (1.6%)	28 (3.1%)	43 (4.8%)
Missing data	760 (21.3%)	243 (27.2%)	218 (24.4%)	165 (18.5%)	134 (15.0%)
**Parity**					
Nulliparous	1,936 (53.5%)	451 (49.9%)	456 (50.4%)	493 (54.5%)	536 (59.3%)
Multiparous	1,680 (46.5%)	453 (50.1%)	448 (49.6%)	411 (45.5%)	368 (40.7%)
**Education**					
Compulsory and lower	154 (4.3%)	48 (5.4%)	40 (4.5%)	31 (3.5%)	35 (3.9%)
High school or equivalent	310 (8.7%)	101 (11.3%)	70 (7.8%)	67 (7.5%)	72 (8.1%)
Graduate or higher	2,864 (80.3%)	664 (74.5%)	710 (79.6%)	736 (82.5%)	754 (84.6%)
Missing data	238 (6.7%)	78 (8.8%)	72 (8.1%)	58 (6.5%)	30 (3.4%)
**Trimester at ferritin test**					
1st trimester	1,008 (28.3%)	32 (3.6%)	74 (8.3%)	277 (31.1%)	625 (70.1%)
2nd trimester	1,416 (39.7%)	347 (38.9%)	423 (47.4%)	425 (47.6%)	221 (24.8%)
3rd trimester	1,142 (32.0%)	512 (57.5%)	395 (44.3%)	190 (21.3%)	45 (5.1%)
**Fe supplements use**					
Non-user	2,421 (67.9%)	580 (65.1%)	583 (65.4%)	603 (67.6%)	655 (73.5%)
Iron supplements	1,145 (32.1%)	311 (34.9%)	309 (34.6%)	289 (32.4%)	236 (26.5%)
**Neonatal characteristic**					
**Gender**					
Male	1,914 (53.7%)	480 (53.9%)	457 (51.2%)	488 (54.7%)	489 (54.9%)
Female	1,652 (46.3%)	411 (46.1%)	435 (48.8%)	404 (45.3%)	402 (45.1%)

Q, quartile.

In the adjusted models, with an increase in Ln-ferritin concentration per unit, the adjusted OR of LBW was 1.78 (95% CI 1.37–2.32) while the OR was 2.05 (1.27–3.32) for LBW (term) (Table 2). For those women whose ferritin concentration were in the highest quartile, the OR was 3.63 (1.52–8.68; *p*-trend < 0.001) for LBW and 5.50 (1.21–25.02; *p*-trend = 0.004) for LBW (term). The ORs of LBW and LBW (term) categories were increased across quartiles of ferritin concentration and the associations were significant in each quartile compare to the lowest quartile. The adjusted OR of LBW (preterm) was 1.64 (95% CI 1.21–2.24) for each additional unit increase of Ln-ferritin concentration. For those women whose ferritin concentration were in the highest quartile, which was suggestively significant associated with LBW (preterm), the OR was 2.46 (0.90–6.76; *p*-trend = 0.01). In the stratified analysis for pregnancy trimesters, the *p* trend for adjusted ORs of LBW was significant in the 2nd (*p*-trend = 0.003) and the last trimesters (*p*-trend < 0.001). In the middle trimester, the OR of LBW of whose ferritin concentration were in the highest quartile was 3.49 (1.54, 7.95). The OR for whose ferritin concentration in the highest quartile of in the last trimester was 26.99 (10.64, 68.45).

**Table 2:** Risk of low birthweight and subcategories associated with maternal serum ferritin concentration.

**Variables**	**OR (95% CI) for ferritin concentration**					***p-*trend**
	**Q1 (*n* = 891) ≤ 12.10 ng/mL**	**Q2 (*n* = 892) 12.10–24.35 ng/mL**	**Q3 (*n* = 892) 24.35–50.61 ng/mL**	**Q4 (*n* = 891) ≥50.61 ng/mL**	**Per unit^†^(*n* = 3,566)**	
**Low birthweight**						
*n* (%)	36 (4.0%)	70 (7.8%)	96 (10.8%)	98 (11.0%)	300 (8.4%)	
Crude	Ref. (OR = 1)	**2.74 (1.29, 5.84)** *	**3.01 (1.42, 6.34)** *	**3.32 (1.61, 6.83)** *	**1.53 (1.20. 1.95)**	**0.001**
Adjusted^‡^	Ref. (OR = 1)	**2.14 (1.03, 4.47)** *	**3.13 (1.47, 6.69)** *	**3.63 (1.52, 8.68)** *	**1.78 (1.37, 2.32)**	**< 0.001**
**Low birthweight (term)**						
*n* (%)	3 (< 1%)	9 (1.0%)	10 (1.1%)	15 (1.7%)	37 (1.0%)	
Crude	Ref. (OR = 1)	2.99 (0.81, 11.10)	**3.23 (0.88, 11.74)** ^**Δ**^	**4.87 (1.40, 16.91)** *	**1.61 (1.13, 2.28)**	**0.009**
Adjusted^‡^	Ref. (OR = 1)	**7.97 (0.96, 66.00)** ^**Δ**^	**7.53 (0.80, 70.56)** ^**Δ**^	**5.50 (1.21, 25.02)** *	**2.05 (1.27, 3.32)**	**0.004**
**Low birthweight (preterm)**						
*n* (%)	15 (1.7%)	26 (2.9%)	38 (4.3%)	34 (3.8%)	113 (3.2%)	
Crude	Ref. (OR = 1)	**2.47 (0.96, 6.41)** ^**Δ**^	**2.78 (1.03, 7.51)** *	**2.47 (0.91, 6.74)** ^**Δ**^	**1.39 (0.98, 1.97)** ^**Δ**^	**0.088** ^**Δ**^
Adjusted^‡^	Ref. (OR = 1)	1.59 (0.70, 3.60)	**2.66 (1.17, 6.02)** *	**2.46 (0.90, 6.76)** ^**Δ**^	**1.64 (1.21, 2.24)**	**0.011** *

Q, quartile.^†^Per unit increase in the natural logarithm transformed serum ferritin concentration (ng/mL).^‡^Adjusted for iron supplements in pregnancy, maternal age at delivery, parity, pre-pregnancy body-mass index, hypertensive disorders in pregnancy, gestational diabetes mellitus, education, and infant sex. ^Δ^*p* < 0.1; *p < 0.05. OR values in bold indicate statistically significance marginally significant.

The adjusted OR of SGA was 1.87 (95% CI 1.38–2.54) with an increase in Ln-ferritin concentration per unit in the adjusted models, and was 3.82 (1.43–10.21; *p*-trend < 0.001) for women whose ferritin level were in the highest quartile group. The ORs of SGA were increased across quartiles of ferritin concentration and the associations were significant in each quartile while the lowest quartile as reference. For the SGA category, increased ferritin concentration posed a greater threat to the occurrence of severe SGA, resulting in OR 2.30 (1.47–3.61) with an increase in Ln-ferritin levels per unit, an OR of 14.07 (1.34–147.77; *p*-trend = 0.001) for women whose ferritin were in the highest quartile (Table 3). In the stratified analyses for pregnancy trimesters, the *p*-trend for adjusted ORs of LBW was significant in the 2nd trimester (*p*-trend = 0.003) and 3rd trimester (*p*-trend < 0.001). In the 2nd trimester, the OR of SGA for women whose ferritin concentration were in the highest quartile was 3.81 (1.50, 9.66) and *p* trend was 0.002 (Supplementary Table S1).

**Table 3:** Risk of small for gestational age and subcategories associated with maternal ferritin concentration.

**Variables**	**OR (95% CI) for ferritin concentration**					***p-*trend**
	**Q1 (*n* = 891) ≤ 12.10 ng/mL**	**Q2 (*n* = 892) 12.10-24.35 ng/mL**,	**Q3 (*n* = 892) 24.35-50.61 ng/mL**	**Q4 (*n* = 891) ≥50.61 ng/mL**	**Per unit^†^(*n* = 3,566)**	
**SGA**						
*n* (%)	14 (1.6%)	25 (2.8%)	26 (2.9%)	30 (3.4%)	95 (2.7%)	
Crude	Ref. (OR = 1)	**1.79 (0.92, 3.47)** ^**Δ**^	**1.75 (0.90, 3.40)** ^**Δ**^	**2.06 (1.08, 3.93)** *	**1.26 (1.01, 1.56)**	**0.047**
Adjusted^‡^	Ref. (OR = 1)	**2.42 (1.08, 5.43)** *	**3.32 (1.40, 7.86)** *	**3.82 (1.43, 10.21)** *	**1.87 (1.38, 2.54)**	**< 0.001**
**Severe SGA**						
*n* (%)	4 (< 1%)	10 (1.1%)	15 (1.7%)	12 (1.3%)	41 (1.1%)	
Crude	Ref. (OR = 1)	2.48 (0.77, 7.95)	**3.50 (1.15, 10.65)** *	**2.77 (0.88, 8.69)** ^**Δ**^	**1.33 (0.96, 1.85)**	**0.086**
Adjusted^‡^	Ref. (OR = 1)	**8.51 (1.06, 68.36)** *	**26.69 (3.06, 232.56)** *	**14.07 (1.34, 147.77)** *	**2.30 (1.47, 3.61)**	**0.001**
**Mild SGA**						
*n* (%)	10 (1.1%)	15 (1.7%)	11 (1.2%)	18 (2.0%)	54 (1.5%)	
Crude	Ref. (OR = 1)	1.48 (0.66, 3.33)	**1.03 (0.43, 2.46)** ^**Δ**^	1.73 (0.79, 3.80)	1.19 (0.90, 1.58)	0.274
Adjusted^‡^	Ref. (OR = 1)	1.60 (0.63, 4.10)	1.15 (0.40, 3.34)	2.57 (0.81, 8.17)	**1.53 (1.02, 2.30)**	**0.024**

Q, quartile.^†^Per unit increase in the natural logarithm transformed maternal serum ferritin concentration (ng/mL).^‡^Adjusted for iron supplements in pregnancy, maternal age at delivery, parity, pre-pregnancy body-mass index, hypertensive disorders in pregnancy, gestational diabetes mellitus, education, and infant sex. ^Δ^*p* < 0.1; *p < 0.05. OR values in bold indicate statistically significance marginally significant.

**Figure 2:**
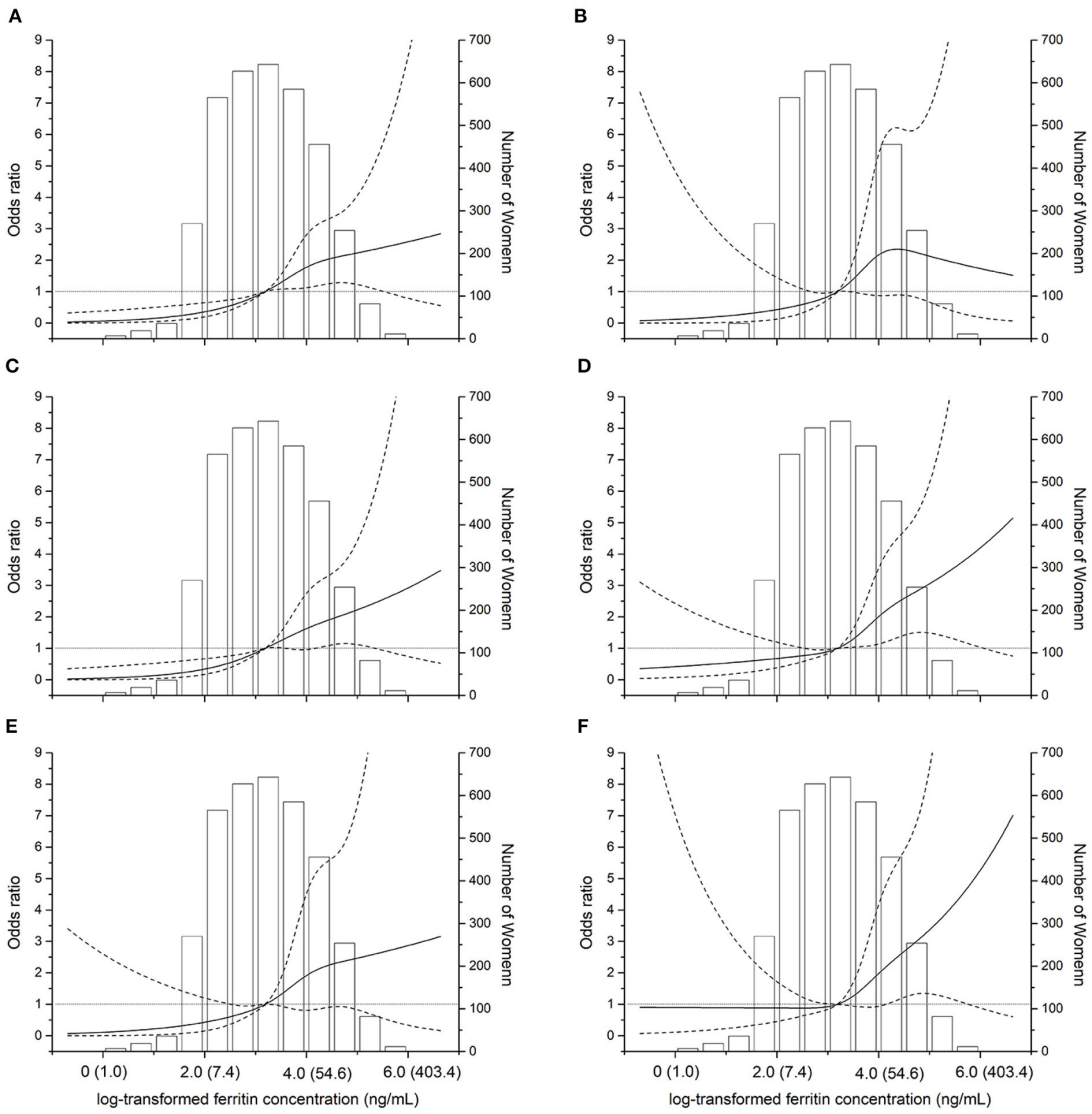
Dose–response relationships between maternal ferritin concentrations and the risk of adverse birth outcomes. The restricted cubic spline for the association between serum ferritin and incident of LBW **(A)**. LBW (term) **(B)**. LBW (preterm) **(C)**. SGA **(D)**. Severe SGA **(E)**. Mild SGA **(F)**. The solid lines represent odds ratios (ORs) based on restricted cubic splines for the log-transformed levels of maternal ferritin, the dotted lines represent the confidence interval. The bars represent histograms of maternal ferritin concentration distribution among the total population. The numbers in parentheses show the maternal concentrations before log-transformation. The horizontal gray line represents OR = 1.

Positive associations of maternal ferritin levels with the risk of birthweight outcomes were observed in the studied population, and the ORs were increasing with higher quartile groups of ferritin level of birthweight outcomes. Spline regression analysis shows significant linear associations for serum Ln-ferritin concentrations and risk of LBW (*p* = 0.005), LBW (term) (*p* = 0.04), and LBW (preterm) (*p* < 0.0001), small for gestational age (*p* = 0.0006), mild SGA (*p* = 0.03), and severe SGA (*p* = 0.02) (Figure 2), which indicates positive associations between maternal ferritin concentrations and ORs of LBW and SGA with a linear manner.

The adjusted ORs of LBW and SGA restricted to women who were without gestational diabetes and hypertension disorders, were lower based on the adjusted model in the sensitivity analyses, the estimated results and statistical significance are consistent with the preliminary analysis (Supplementary Table S2).

Additionally, the adjusted OR (95% CI) of LBW and SGA among women using supplemental iron was 0.56 (0.38, 0.85) and 0.65 (0.40, 1.05) compared with non-users, respectively (Supplementary Table S3). The present study suggests that Fe supplementation use for pregnant women in the high ferritin group were not associated with higher risk of LBW.

## Discussion

This is the first and largest retrospective cohort study to investigate the relationship between maternal ferritin concentration and birthweight among mainland Chinese pregnant women. Our results showed that the risk of low birthweight is significantly increased in pregnant women with higher ferritin concentration. We further investigated the dose–response relationship between maternal ferritin and the risk of low birthweight for the first time. In this study, it was suggested that elevated maternal ferritin levels during pregnancy were associated with a higher risk of LBW and SGA in a linear dose–response manner.

The positive associations between concentrations of serum ferritin, used in medical practice to indicate iron storage and anemia status, with LBW and SGA in our study were in accordance with previous studies (6, 7, 15–19). Goldenberg et al. found that participants in high ferritin group was associated with lower birthweight when the low ferritin group was as reference among 580 black women (6). Hubel et al. demonstrated that elevated maternal ferritin levels were increased 3-fold risk of intrauterine growth restriction (IUGR) (18). Akkurt et al. reported that the maternal ferritin level was higher in the IUGR group than in the appropriate for gestational age (AGA) group (59 ng/mL vs. 32 ng/mL, *p* < 0.001) (16). A prospective cohort study was conducted among 573 pregnant women which discovered a negative association between high plasma ferritin and low birthweight (15). The fact that previous studies were in small sample sizes with < 600 participants, our data from a large retrospective cohort study provide strong scientific evidence in a Chinese population. In the present study, the median serum ferritin concentration (median = 24 ng/mL) was comparable to values from previous studies, from which the mean/median levels ranged from 18 to 43 ng/mL across pregnancy (6, 15, 17, 20, 21). As a result, the consistency between studies indicates the adverse association between maternal ferritin concentrations and birthweight effects persisting in Chinese and other ethnic populations. Of note, the positive association of ferritin and LBW and SGA was observed in the 2nd and/or 3rd trimesters in the stratified analysis (Supplementary Table S1), and this phenomenon is consistent with previous research (8).

In the present study, we observed an increased risk of LBW and SGA as the maternal ferritin concentration increased in a linear dose-response manner. However, there was a hypothesis that iron exhibits a U-shaped risk of adverse health effect curves similar to other internal nutrients, which was not consistent with the results in our study. This discrepancy is probably have multiple explanations because of the double character of maternal ferritin both as an iron storage marker and acute stage reactant (7, 22), such as oxidative stress (7, 23), inflammation and infection (22, 24), hemoconcentration (7, 25). A higher ferritin concentration in pregnant women was also found to be associated with a higher risk of preterm delivery and gestational diabetes in previous studies, it may add risk of health risk as a whole (26).

The present study suggests that Fe supplementation use is beneficial to birthweight-related outcomes, however, for pregnant women without iron deficiency may be not helpful for reducing the risk of LBW. In fact, the evidence supporting the guidance for universal supplementation of iron among gestational women has been controversial (8). In the context of a high global anemia rate in pregnant women, iron deficiency accounts for 20–50% of anemia occurrences (27, 28). Comparative evidence suggests that iron supplementation improves hematologic biomarkers such as hemoglobin and ferritin (7, 29). However, there are also studies reported that routine iron supplementation in non-anemic gestational woman has no benefit on pregnancy outcomes, and caution is advised against Fe supplementation already (8). The discrepancy was possibly due to different body iron stores in the studied populations. Thus, iron supplementation among women with high maternal ferritin was not associated with significantly decreased risk of LBW.

There are notable strengths of the present study. For the first time, our study revealed whether maternal serum ferritin was associated with an increased risk of birthweight related outcomes in a retrospective cohort study based on a large sample size among Chinese population. We disclosed that as maternal ferritin concentrations rise, it would reversely associate with fetal weight growth that add risks of LBW and SGA, the second and third trimesters should be highly focused on. Thus, an assessment of iron status during pregnancy is needed to make appropriate individualized iron supplementation recommendations to prevent negative outcomes because of maternal iron deficiency and iron overload.

Nevertheless, this study also has potential limitations. First, we only used maternal ferritin as a marker of maternal iron status among the studied pregnant women. Among markers of iron status, serum ferritin has been the most used markers in epidemiologic studies (8); however, including other markers (transferrin et al.) would be more comprehensive for interpreting the results. Second, information on the intake dose of iron supplements and diet was absent. Third, we did not have a biological sample of the studied population, which could be further tested for oxidative stress and inflammation status.

In conclusion, maternal ferritin levels during pregnancy may provide an additional predictor for distinguishing fetuses with potential poor birthweight related outcomes. Moreover, high ferritin values should also be monitored by prenatal care due to adverse perinatal outcome. Further exploration should be conducted to ensure maternal ferritin thresholds and iron supplement doses.

## Data availability statement

The original contributions presented in the study are included in the article/Supplementary materials, further inquiries can be directed to the corresponding author/s.

## Author contributions

YZZ and JHM initiated the project. YT conducted the research and analyzed the data and wrote the initial draft of the paper. YS extract and review the data. JWK, JL, JD, FW, DX, XLQ, JJG, YJL, and CW contributed to revisions. All authors approved the final manuscript.

## Funding

This study was supported by Hubei Science and Technology Plan (2017ACB640), China Postdoctoral Science Foundation (2022M712454).

## Conflict of interest

The authors declare that the research was conducted in the absence of any commercial or financial relationships that could be construed as a potential conflict of interest.

## Publisher's note

All claims expressed in this article are solely those of the authors and do not necessarily represent those of their affiliated organizations, or those of the publisher, the editors and the reviewers. Any product that may be evaluated in this article, or claim that may be made by its manufacturer, is not guaranteed or endorsed by the publisher.
