# Supplementary material for: Association of low birthweight and small for gestational age with maternal ferritin levels: A retrospective cohort study in China
Source: Front Nutr. 2022 Oct 10;9:1002702. doi: 10.3389/fnut.2022.1002702 (PMC9589249; doi:10.3389/fnut.2022.1002702)
Supplement: Supplementary file 1 [file Data_Sheet_1.docx]

Association of low birthweight and small for gestational age with maternal serum ferritin levels: a retrospective cohort study in China

**Yun Tao^1,2,3†^, Jiawei Kang^1,2,3†^, Juan Liu^1,2,3^, Jie Duan^1,2,3^, Fang Wang^1,2,3^, Yue Shi^4^, Yujuan Li^1,2,3^, Cheng Wang^1,2,3^, Dan Xu^1,2,3^, Xinlan Qu^1,2^****^,3^, Juanjuan Guo****^1,2,3^, Jianhong Ma^1,2,3*^, Yuanzhen Zhang^1,2,3*^**

^1^ Department of Obstetrics and Gynecology, Zhongnan Hospital of Wuhan University, Wuhan, China.

^2^ Hubei Clinical Research Center for Prenatal Diagnosis and Birth Health, Wuhan, China.

^3^ Wuhan Clinical Research Center for Reproductive Science and Birth Health, Wuhan, China.

^4^ Information Center, Zhongnan Hospital of Wuhan University, Wuhan, China.

^*^ Correspondence:

Yuanzhen Zhang, Department of Obstetrics and Gynecology; Zhongnan Hospital of Wuhan University; Hubei Clinical Research Center for Prenatal Diagnosis and Birth Health; Wuhan Clinical Research Center for Reproductive Science and Birth Health, Wuhan, China. Email: [zhangyuanzhen@whu.edu.cn](mailto:zhangyuanzhen@whu.edu.cn);

Jianhong Ma, Department of Obstetrics and Gynecology; Zhongnan Hospital of Wuhan University; Hubei Clinical Research Center for Prenatal Diagnosis and Birth Health; Wuhan Clinical Research Center for Reproductive Science and Birth Health, Wuhan, China. Email: [majianh2005@163.com](mailto:majianh2005@163.com)

^†^ These authors contributed equally to this work.

Table S1 Associations between maternal ferritin concentrations during pregnancy with LBW and SGA stratified by trimester^‡^

|  | Vaiables | N | Unajusted OR | | Ajusted OR | |  |
| --- | --- | --- | --- | --- | --- | --- | --- |
| LBW | **First trimester** | 1008 |  | |  | |  |
|  | Quantile 1 | 5 (<1%) | 1.00 (Ref.) | | 1.00 (Ref.) | |  |
|  | Quantile 2 | 12 (1.2%) | 2.47 (0.86, 7.12) | | 2.14 (0.72, 6.36) | |  |
|  | Quantile 3 | 7 (<1%) | 1.41 (0.44, 4.51) | | 1.75 (0.52, 5.87) | |  |
|  | Quantile 4 | 11 (1.1%) | 2.25 (0.77, 6.59) | | 2.34 (0.77, 7.18) | |  |
|  | *P* trend |  | 0.27 | | 0.33 | |  |
|  | **Second trimester** | 1416 |  | |  | |  |
|  | Quantile 1 | 8 (<1%) | 1.00 (Ref.) | | 1.00 (Ref.) | |  |
|  | Quantile 2 | 16 (1.1%) | 2.05 (0.87, 4.86) | | 1.96 (0.82, 4.66) | |  |
|  | Quantile 3 | 15 (1.1%) | 1.92 (0.81, 4.60) | | 1.70 (0.70, 4.13) | |  |
|  | Quantile 4 | 29 (2.0%) | **3.87 (1.74, 8.59) ^*^** | | **3.49 (1.54, 7.95) ^*^** | |  |
|  | *P* trend |  | **0.001** | | **0.003** | |  |
|  | **Third trimester** | 1142 |  | |  | |  |
|  | Quantile 1 | 5 (<1%) | 1.00 (Ref.) | | 1.00 (Ref.) | |  |
|  | Quantile 2 | 106 (9.3%) | **32.98 (13.19, 82.44) ^*^** | | **34.91 (13.87, 87.87) ^*^** | |  |
|  | Quantile 3 | 97 (8.5%) | **28.74 (11.48, 71.94) ^*^** | | **28.89 (11.51, 72.53) ^*^** | |  |
|  | Quantile 4 | 88 (7.7%) | **25.02 (9.98, 62.73) ^*^** | | **26.99 (10.64, 68.45) ^*^** | |  |
|  | *P* trend |  | **<0.001** | | **<0.001** | |  |
| SGA | **First trimester** | 1008 |  | |  | |  |
|  | Quantile 1 | 1 (<1%) | 1.00 (Ref.) | | 1.00 (Ref.) | |  |
|  | Quantile 2 | 5 (<1%) | 5.08 (0.59, 43.80) | | 3.97 (0.45, 34.93) | |  |
|  | Quantile 3 | 4 (<1%) | 4.05 (0.45, 36.48) | | 4.01 (0.43, 37.48) | |  |
|  | Quantile 4 | 6 (<1%) | 6.12 (0.73, 51.22) | | 4.36 (0.50, 38.10) | |  |
|  | *P* trend |  | 0.11 | | 0.17 | |  |
|  | **Second trimester** | 1416 | |  | |  |  |
|  | Quantile 1 | 6 (<1%) | 1.00 (Ref.) | | 1.00 (Ref.) | |  |
|  | Quantile 2 | 10 (<1%) | 1.69 (0.61, 4.70) | | 1.71 (0.61, 4.78) | |  |
|  | Quantile 3 | 9 (<1%) | 1.52 (0.54, 4.32) | | 1.37 (0.47, 3.98) | |  |
|  | Quantile 4 | 22 (1.6%) | **3.85 (1.54, 9.62) ^*^** | | **3.81 (1.50, 9.66) ^*^** | |  |
|  | *P* trend |  | **0.002** | | **0.002** | |  |
|  | **Third trimester** | 1142 | |  | |  |  |
|  | Quantile 1 | 7 (<1%) | 1.00 (Ref.) | | 1.00 (Ref.) | |  |
|  | Quantile 2 | 1 (<1%) | 0.14 (0.02, 1.14) | | 0.14 (0.02, 1.13) | |  |
|  | Quantile 3 | 3 (<1%) | 0.42 (0.11, 1.64) | | 0.46 (0.11, 1.85) | |  |
|  | Quantile 4 | 8 (<1%) | 1.15 (0.41, 3.21) | | 1.13 (0.39, 3.29) | |  |
|  | *P* trend |  | 0.50 | | 0.52 | |  |

‡ Adjusted for iron supplements in pregnancy, maternal age at delivery, parity, pre-pregnancy body-mass index, hypertensive disorders in pregnancy, gestational diabetes mellitus, education, and infant sex.

Table S2 Risk of adverse birth outcomes associated with maternal serum ferritin concentrations restricted to women without hypertensive disorders in pregnancy and gestational diabetes mellitus

| Variables | OR (95% CI) for ferritin concentration | | | | | *p-*trend |
| --- | --- | --- | --- | --- | --- | --- |
|  | Q1 (n=726)  ≤11.57ng/mL | Q2 (n=727)  11.57-22.75ng/mL | Q3 (n=726)  22.75-47.93ng/mL | Q4 (n=726)  ≥47.93ng/mL | Per unit^†^（n=2905） |  |
| Low birthweight | | | | | | |
| n (%) | 16 (2.20%) | 25 (3.44%) | 37 (5.10%) | 44 (6.06%) |  |  |
| Crude | Ref. (OR=1) | 1.58 (0.84, 2.99) | **1.98 (1.13, 3.45) ^*^** | **2.86 (1.60, 5.12) ^*^** | **1.43 (1.19, 1.73)** | **<0.001** |
| Adjusted^‡^ | Ref. (OR=1) | 1.53 (0.81, 2.91) | **1.97 (1.12, 3.44) ^*^** | **2.89 (1.61, 5.20) ^*^** | **1.43 (1.18, 1.74)** | **<0.001** |
| SGA | | | | | | |
| n (%) | 10 (1.38%) | 18 (2.48%) | 18 (2.48%) | 28 (3.86%) |  |  |
| Crude | Ref. (OR=1) | 1.82 (0.83, 3.97) | **1.82 (0.90, 3.69) ^△^** | **2.87 (1.38, 5.96) ^*^** | **1.38 (1.08, 1.75)** | **0.004** |
| Adjusted^‡^ | Ref. (OR=1) | 1.73 (0.79, 3.78) | 1.74 (0.86, 3.53) | **2.77 (1.32, 5.83) ^*^** | **1.33 (1.05, 1.70)** | **0.008** |

Q=quartile. † Per unit increase in the natural logarithm transformed maternal serum ferritin concentration (ng/mL). ‡ Adjusted for iron supplements in pregnancy, maternal age at delivery, parity, pre-pregnancy body-mass index, hypertensive disorders in pregnancy, gestational diabetes mellitus, education, and infant sex.

Table S3 Associations between maternal ferritin concentrations during pregnancy with LBW and SGA stratified by Fe supplements^‡^

| Vaiables | | N (%) | Unajusted OR | Ajusted OR |
| --- | --- | --- | --- | --- |
| LBW | Nonusers | 119 (4.9%) | 1.00 (Ref.) | 1.00 (Ref.) |
|  | Fe supplement user | 31 (1.3%) | **0.54 (0.36, 0.80) ^*^** | **0.56 (0.38, 0.85) ^*^** |
| SGA | Nonusers | 72 (6.3%) | 1.00 (Ref.) | 1.00 (Ref.) |
|  | Fe supplement user | 23 (2.0%) | **0.67 (0.42, 1.08) ^△^** | **0.65 (0.40, 1.05) ^△^** |

‡ Adjusted for iron supplements in pregnancy, maternal age at delivery, parity, test trimester, hypertensive disorders in pregnancy, and gestational diabetes mellitus.


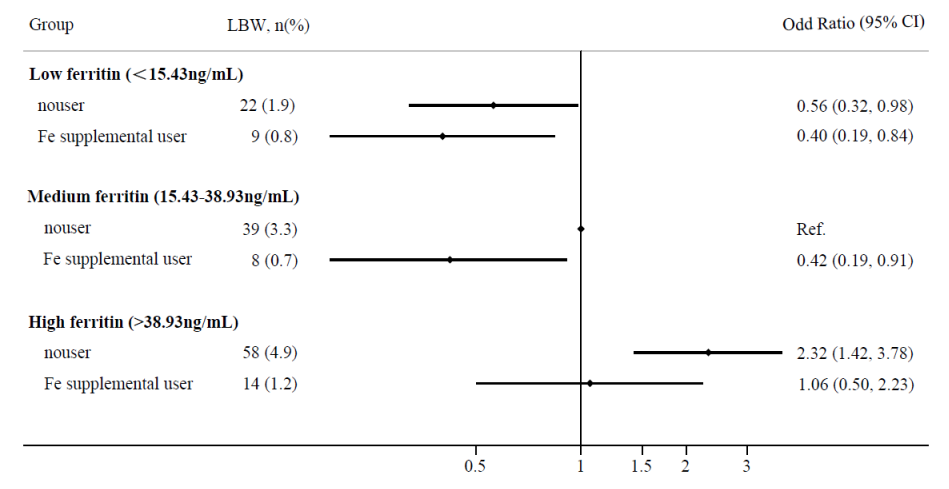


Figure S1 Association of maternal ferritin concentration and iron supplement use with LBW. ^‡^

‡ Adjusted for iron supplements in pregnancy, maternal age at delivery, parity, test trimester, hypertensive disorders in pregnancy, and gestational diabetes mellitus.
